# Detection of functional and structural brain alterations in female schizophrenia using elastic net logistic regression

**DOI:** 10.1007/s11682-021-00501-z

**Published:** 2021-07-27

**Authors:** Ying Wu, Ping Ren, Rong Chen, Hong Xu, Jianxing Xu, Lin Zeng, Donghui Wu, Wentao Jiang, NianSheng Tang, Xia Liu

**Affiliations:** 1 Key Lab of Statistical Modeling and Data Analysis of Yunnan, Yunnan University, Kunming, China; 2 Lab of Brain Health Assessment and Research, Shenzhen Mental Health Center, Shenzhen, China; 3 Department of Geriatric Psychiatry, Shenzhen Kangning Hospital, Shenzhen, China; 4 Female Ward of Acute Psychiatric Department, Shenzhen Kangning Hospital, Shenzhen, China; 5 Neuropsychiatry Imaging Center, Shenzhen Mental Health Center, Shenzhen, China; 6 Department of Radiology, Shenzhen Kangning Hospital, Shenzhen, China; 7 School of Mental Health, Jining Medical University, Jining, China

**Keywords:** Schizophrenia, Resting-state functional magnetic resonance imaging, Elastic net regression, Amplitude of low frequency fluctuation, Gray matter volume

## Abstract

Neuroimaging technique is a powerful tool to characterize the abnormality of brain networks in schizophrenia. However, the neurophysiological substrate of schizophrenia is still unclear. Here we investigated the patterns of brain functional and structural changes in female patients with schizophrenia using elastic net logistic regression analysis of resting-state functional magnetic resonance imaging data. Data from 52 participants (25 female schizophrenia patients and 27 healthy controls) were obtained. Using an elastic net penalty, the brain regions most relevant to schizophrenia pathology were defined in the models using the amplitude of low-frequency fluctuations (ALFF) and gray matter, respectively. The receiver operating characteristic analysis showed reliable classification accuracy with 85.7% in ALFF analysis, and 77.1% in gray matter analysis. Notably, our results showed eight common regions between the ALFF and gray matter analyses, including the Frontal-Inf-Orb-R, Rolandic-Oper-R, Olfactory-R, Angular-L, Precuneus-L, Precuenus-R, Heschl-L, and Temporal-Pole-Mid-R. In addition, the severity of symptoms was found positively associated with the ALFF within the Rolandic-Oper-R and Frontal-Inf-Orb-R. Our findings indicated that elastic net logistic regression could be a useful tool to identify the characteristics of schizophrenia -related brain deterioration, which provides novel insights into schizophrenia diagnosis and prediction.

**Supplementary Information:**

The online version contains supplementary material available at 10.1007/s11682-021-00501-z.

## Introduction

Schizophrenia (SZ) is a severe mental disorder characterized by hallucinations, delusions and cognitive impairments. So far, the diagnosis of SZ is mainly based on the Diagnostic and Statistical Manual of Mental Disorders 5 (DSM-5) or the International Classification of Diseases (ICD) (First, 2013; van Os & Kapur, 2009). In recent years, neuroimaging technique has been widely used in studying neurobiological changes in the brain in multiple psychiatric disorders, which provides useful biomarkers in pre-clinical research and clinical diagnosis. Compared with healthy controls (HC), accumulated evidence from magnetic resonance imaging (MRI) studies have shown widespread brain dysfunction in SZ patients, including the frontal cortex, temporal lobe and subcortical regions (Mwansisya et al., 2017). However, it is still unclear about the neurophysiological substrate of SZ, and how to accurately diagnose and predict SZ using regional features derived from imaging data.

Resting-sate functional MRI (rs-fMRI) measures intrinsic regional activity and functional connectivity of brain in the absence of external tasks. Biswal et al. found that the spontaneous low-frequency oscillations (LFO) of blood-oxygen-level-dependent (BOLD) signals measured in rs-fMRI are physiologically meaningful (Biswal et al., 1995), and the LFO has been successfully applied in studying neural substrates of brain dysfunction and psychiatric disorders (Woodward & Cascio, 2015). Different from functional connectivity, the amplitude of low-frequency fluctuations (ALFF) measures the intensity of resting-state BOLD signals in the frequency range from 0.01 to 0.08 Hz (Zang et al., 2007). Previous studies have found significant changes of ALFF in multiple brain regions in SZ (Hoptman et al., 2010; Yu et al., 2014). In a large and multisite sample of SZ patients, poorer cognitive functions were found associated with lower fractional ALFF in multiple brain regions, including the anterior cingulate cortex, dorsolateral prefrontal cortex, and posterior parietal cortex (Fryer et al., 2015). In addition to brain functional changes, SZ patients exhibited widespread brain structural changes as well, such as gray matter (GM) loss and white matter disconnection (Gupta et al., 2015; Najjar & Pearlman, 2015). Although the neuroimaging findings indicate the functional and structural changes could be potential biomarkers for SZ diagnosis, the neural substrate of SZ is still under-investigated.

In recent years, there is a growing effort devoted to develop statistical methods for clinical diagnosis with greater accuracy and efficiency. Multiple linear regression and stepwise regression approaches are widely used in neuroimaging data analysis to characterize the brain alterations in psychiatric disorders (Agosta et al., 2012; Sheline & Raichle, 2013). However, these classical regression models have significant limitations in dealing with neuroimaging data. For example, features from rs-fMRI data are often correlated across different regions, and linear regression approaches are known to be highly sensitive to collinearity. Therefore, regularization techniques have been established to deal with multidimensional and multicollinear issues in neuroimaging data, such as ridge regression and the Lasso regression (Bunea et al., 2011; Guo et al., 2018; Kashyap et al., 2019). Recently, an elastic net penalty, which combines the Lasso and ridge regression penalty, was developed to solve dimension reduction and feature selection problem by Zou et al. (Zou & Hastie, 2005). The elastic net logistic regression could be used to effectively differentiate patients from controls, which has been used in Alzheimer’s disease and other brain diseases (de Vos et al., 2016; Teipel et al., 2016). Zhu and his colleagues applied a non-negative elastic-net based method to examine the altered resting-state brain functional connectivity in SZ patients (Zhu et al., 2018). Using support vector machine, Savio et al. compared multiple fMRI measures for identifying SZ in a large public database and reported 60%—70% accuracy with ALFF/fALFF (Savio & Graña, 2015). Another study applied structural MRI data for SZ diagnosis to compare different machine learning algorithms including elastic net, and showed 75% prediction accuracy averaging over classifiers (Salvador et al., 2017). To the best of our knowledge, there has been no study applying elastic net logistic regression to comparing brain functional and structural alterations in SZ using ALFF and gray matter measures.

In the present study, we investigated the neurobiological changes in the brain of female SZ patients by comparing brain functional and structural patterns. Elastic net logistic regression analyses were applied to define SZ-related brain regions in ALFF and GM measures, respectively. The receiver operating characteristic (ROC) curves were used to measure the classification sensitivity and specificity of the models. We hypothesized that the elastic net logistic regression would successfully identify the SZ-related brain alterations and discriminate between SZ and HC groups. Additionally, we speculate there would be several overlapped regions between ALFF and GM analysis, which may play key roles in the progression of SZ.

## Materials and methods

### Participants

Twenty-nine female SZ patients were recruited from women’s psychiatric unit at the Department of Inpatient in Shenzhen Kangning Hospital, and 31 female HCs were recruited from multiple communities in Shenzhen. All participants were right handed determined by the Edinburgh handedness inventory. In the SZ group, all participants met the DSM-IV criteria for paranoid SZ according to a diagnostic assessment using the Structured Clinical Interview for DSM-IV Patient Edition (SCID-P), and were either medication-naive or unmedicated during past 4 months. In the HC group, participants were examined to exclude those with first-degree relatives having SZ, schizoaffective disorder, or other psychiatric disorders. The demographic information of each participant was collected, including age, education and medical history. For each SZ patients, clinical symptoms were recorded based on the Positive and Negative Syndrome Scale (PANSS). The Positive Symptoms (PANSS-P), Negative Symptoms (PANSS-N), and General Symptoms (PANSS-G) were assessed respectively by two experienced psychiatrists (see Table 1). All participants were free of any significant neurological disease, head trauma, cardiovascular disease, alcohol/substance abuse, pregnancy, or physical illness. The study was approved by the Ethics Committee of Shenzhen Kangning Hospital. Each participant was required to sign a written informed consent form after a full written and verbal explanation of the study.

**Table 1:** Demographic and characteristics of subjects

	SZ (*n* = 25)	HC (*n* = 27)	*p* value
Age	33.3 ± 10.2	32.7 ± 10.7	0.8
Years of education	12.5 ± 3.0	14.7 ± 3.5	0.02*
PANSS-P score	28.1 ± 7.2		
PANSS-N score	18.0 ± 9.4		
PANSS-G score	46.5 ± 12.6		

Data are presented as means ± standard deviations

*Abbreviations*: *SZ* schizophrenia, *HC* healthy control, *PANSS* Positive and negative Syndrome Scale, *P* positive, *N* negative, *G* general

*p < 0.05

### Imaging data acquisition and preprocessing

Imaging data were acquired on a 3.0 T MR system (Discovery MR750 System, GE Healthcare) with an eight-channel phased-array headcoil. The rs-fMRI data were acquired using gradient-echo echo-planar imaging sequence with the following parameters: repetition time (TR) = 2000 ms, echo time (TE) = 25 ms, number of slices = 35, section thickness = 3 mm, intersection gap = 1 mm; matrix = 64 × 64, and spatial resolution = 3.75 × 3.75 × 3 mm^3^. For each participant, the rs-fMRI scanning lasted 420 s with 210 volumes. Then structural images were acquired by using a three-dimensional brain volume imaging sequence that covered the whole brain (TR = 8.2 ms, TE = 3.2 ms, matrix = 256 × 256, section thickness = 1 mm, 136 slices). During the entire scanning, participants were required to close their eyes and relax without falling asleep.

The functional imaging data were preprocessed using DPARSF (Chao-Gan & Yu-Feng, 2010) based on SPM8 (http://www.fil.ion.ucl.ac.uk/spm/). For each participant, the first 10 volumes were excluded to obtain steady-state tissue magnetization. The remaining 200 volumes were corrected for slice timing and head motion, co-registered to their own structural images, and normalized to the Montreal Neurological Institute (MNI) standard space. Then the imaging data were resampled to 3 × 3 × 3 mm, and smoothed using a Gaussian kernel (FWHM = 6 mm). After preprocessing the functional data, 4 SZs and 4 HCs were removed from the formal analysis due to head motion greater than 2 mm or 2 degrees.

### ALFF and GM analysis

After removing the linear trend, a band pass filter (0.01–0.08 Hz) was applied to remove non-biological signals. Ninety regions of interest (ROI) were selected for the following analysis based on the Automated Anatomical Labeling atlas (AAL) (Tzourio-Mazoyer et al., 2002). In the ALFF analysis, the time courses of BOLD signal were converted to frequency domain using the fast Fourier transform. The square root of the power spectrum was then calculated and averaged across 0.01–0.08 Hz for each voxel. The averaged square root was defined as the ALFF value at the given voxel (Zang et al., 2007). To eliminate the whole-brain differences of ALFF across individuals, the resulting ALFF map was converted to z-score by subtracting the global mean and dividing the global standard deviation. Next the averaged ALFF value within each ROI was extracted for logistic regression analysis.

Voxel-based morphometry (VBM) was performed to generate a whole-brain GM map in DPARSF. The structural image of each participant was segmented into GM, white matter and cerebrospinal fluid. Then, a GM template was generated through an iteratively nonlinear registration using DARTEL, a toolbox with a fast diffeomorphic registration algorithm (Ashburner, 2007). The GM template was used for normalizing functional images to MNI space. For each participant, averaged GM value within each ROI was extracted for the following analysis.

### Elastic net logistic regression

**Fig. 1:**
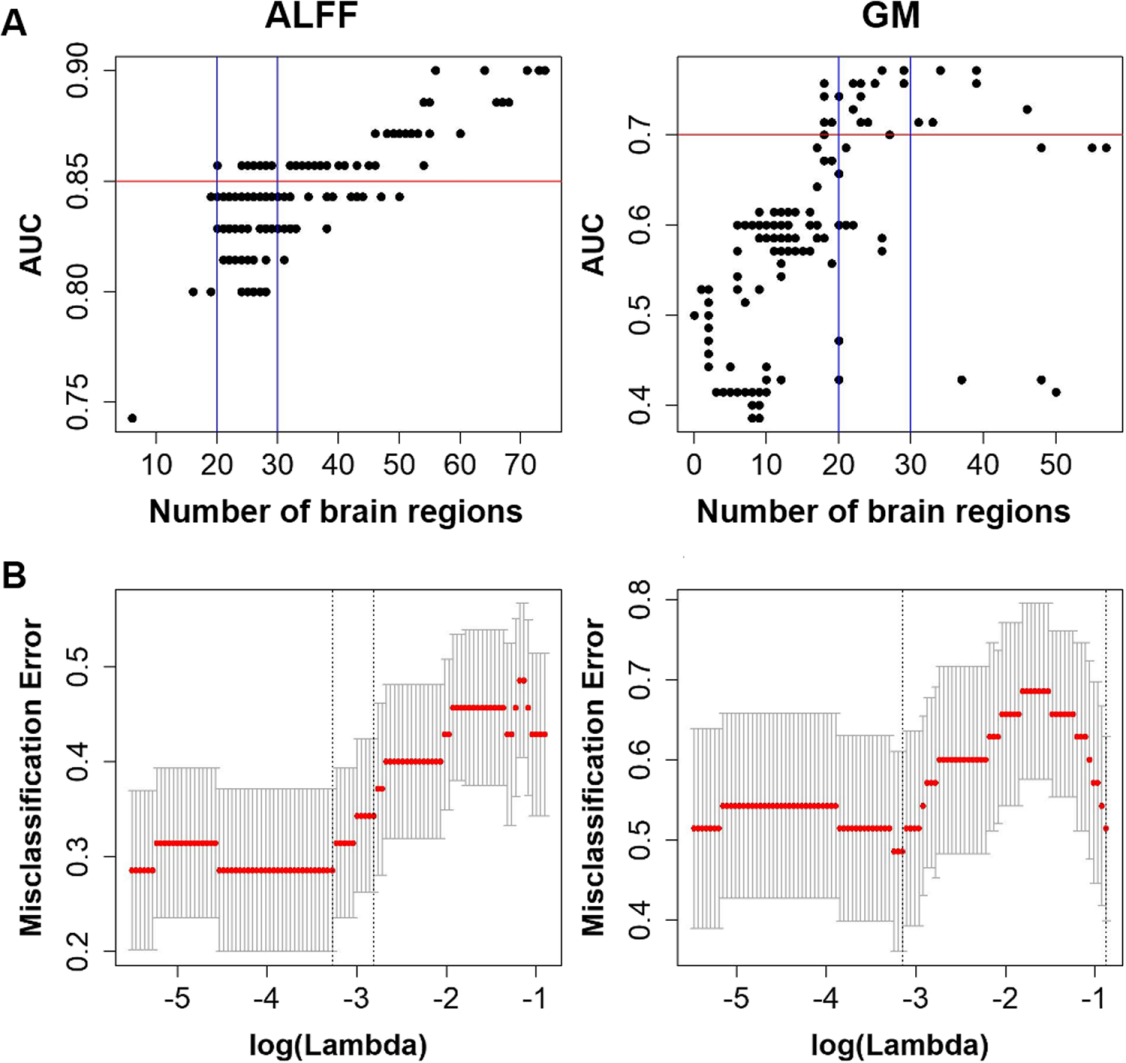
The parameters in logistic regression were defined using elastic net penalty. **A** The scatterplots show the largest AUC values obtained by using 20–30 brain regions in both ALFF and GM analyses. **B** The optimal λ values were determined by the minimum misclassification errors in ALFF (log(λ) = −1.42) and GM (log(λ) = −1.37), respectively. Abbreviations: AUC, area under the curve; GM, gray matter; ALFF, amplitude of low-frequency fluctuations

The features in ALFF and GM measures were used to detect the alterations of brain pattern in SZ, thus we applied logistic regression models with an elastic net penalty using the R package *glmnet* (https://cran.r-project.org/web/packages/glmnet/index.html) and *pROC* (https://cran.r-project.org/web/packages/pROC/index.html). The elastic net logistic regression combined ridge regression and Lasso regression to minimize the loss function (Zou & Hastie, 2005). In the model, α is the mixing parameter between ridge (α = 0) and Lasso (α = 1), and λ indicates the strength of regularization (Friedman et al., 2010). The optimal α value was obtained from the range 0 to 1 based on the ROC curve, and the optimal λ was defined based on minimum misclassification error (see Fig. 1). The detailed information can be found in the results.

To avoid the overfitting problem, we used tenfold cross-validation with 35 participants in the training set (15 SZs and 20 HCs) and 17 participants in the test set (10 SZs and 7 HCs). In ROC analysis, the area under the curve (AUC) was used to assess the classification performance. Sensitivity and specificity were calculated to assess the goodness of prediction in ALFF and GM analyses, respectively. After the most relevant brain regions contributing to SZ prediction were selected, the common regions across the two analyses were chosen to do the following correlation analysis. These common regions were defined as core components characterizing the altered brain network due to SZ pathology.

### Other statistical analysis

Other statistical analysis was conducted in SPSS22. In correlation analysis, one of the subjects was excluded as an outlier due to high PANSS scores. And log-transformation was applied to all PANSS scores to reduce data skewness. Then a partial correlation was used to examine the relationship between brain regions (ALFF and GM) and PANSS scores, controlled for age and education. Bonferroni correction for multiple comparisons was not applied in order to comprehensively present all possible correlations.

## Results

### Demographic analysis

The demographic data for all participants were shown in Table 1. There was a significant difference between SZs and HCs in education (*p* = 0.02), but not in age (*p* = 0.8).

### Elastic net logistic regression in SZ classification

In the elastic net logistic regression model, the two parameters α and λ were defined according to the ROC curve for ALFF and GM measures, respectively. Figure 1A shows the relationships between the number of selected brain regions and AUC values. Given the maximal AUC values, 20–30 brain regions were chosen as the best predictors in both ALFF (AUC = 0.85, α = 0.56) and GM (AUC = 0.70, α = 0.55). Eventually, 27 brain regions were chosen in ALFF analysis, and 26 brain regions were chosen in GM analysis (see Table 2). The optimal λ value was determined by the minimal classification errors in ALFF (λ = 0.038) and GM (λ = 0.043), respectively (see Fig. 1B). In addition, further analyses were applied to compare tenfold cross validation to fivefold, 15-fold and 20-fold, and showed tenfold cross validation giving the optimal parameters in both ALFF and GM analyses (Supplementary Table 1).

**Table 2:** Brain regions associated with SZ according to elastic net logistic regression

ALFF		GM	
AAL No	Brain region	AAL No	Brain region
2	Precentral-R	1	Precentral-L
#16	Frontal-Inf-Orb-R	6	Frontal-Sup-Orb-R
#18	Rolandic-Oper-R	7	Frontal-Mid-L
19	Supp-Motor-Area-L	8	Frontal-Mid-R
20	Supp-Motor-Area-R	14	Frontal-Inf-Tri-R
#22	Olfactory-R	#16	Frontal-Inf-Orb-R
24	Frontal-Sup-Medial-R	#18	Rolandic-Oper-R
34	Cingulum-Mid-R	21	Olfactory-L
35	Cingulum-Post-L	#22	Olfactory-R
36	Cingulum-Post-R	37	Hippocampus-L
38	Hippocampus-R	41	Amygdala-L
39	ParaHippocampal-L	42	Amygdala-R
48	Lingual-R	43	Calcarine-L
51	Occipital-Mid-L	45	Cuneus-L
58	Postcentral-R	53	Occipital-Inf-L
61	Parietal-Inf-L	56	Fusiform-R
#65	Angular-L	60	Parietal-Sup-R
#67	Precuneus-L	62	Parietal-Inf-R
#68	Precuneus-R	#65	Angular-L
71	Caudate-L	#67	Precuneus-L
72	Caudate-R	#68	Precuneus-R
74	Putamen-R	69	Paracentral-Lobule-L
#79	Heschl-L	#79	Heschl-L
80	Heschl-R	84	Temporal-Pole-Sup-R
81	Temporal-Sup-L	87	Temporal-Pole-Mid-L
85	Temporal-Mid-L	#88	Temporal-Pole-Mid-R
#88	Temporal-Pole-Mid-R		

The common regions in both ALFF and GM analyses were marked with #

*Abbreviations*: *SZ* schizophrenia, *ALFF* amplitude of low-frequency fluctuations, *GM* gray matter, *AAL* automated anatomical labeling atlas, *L* left, *R* right

**Fig. 2:**
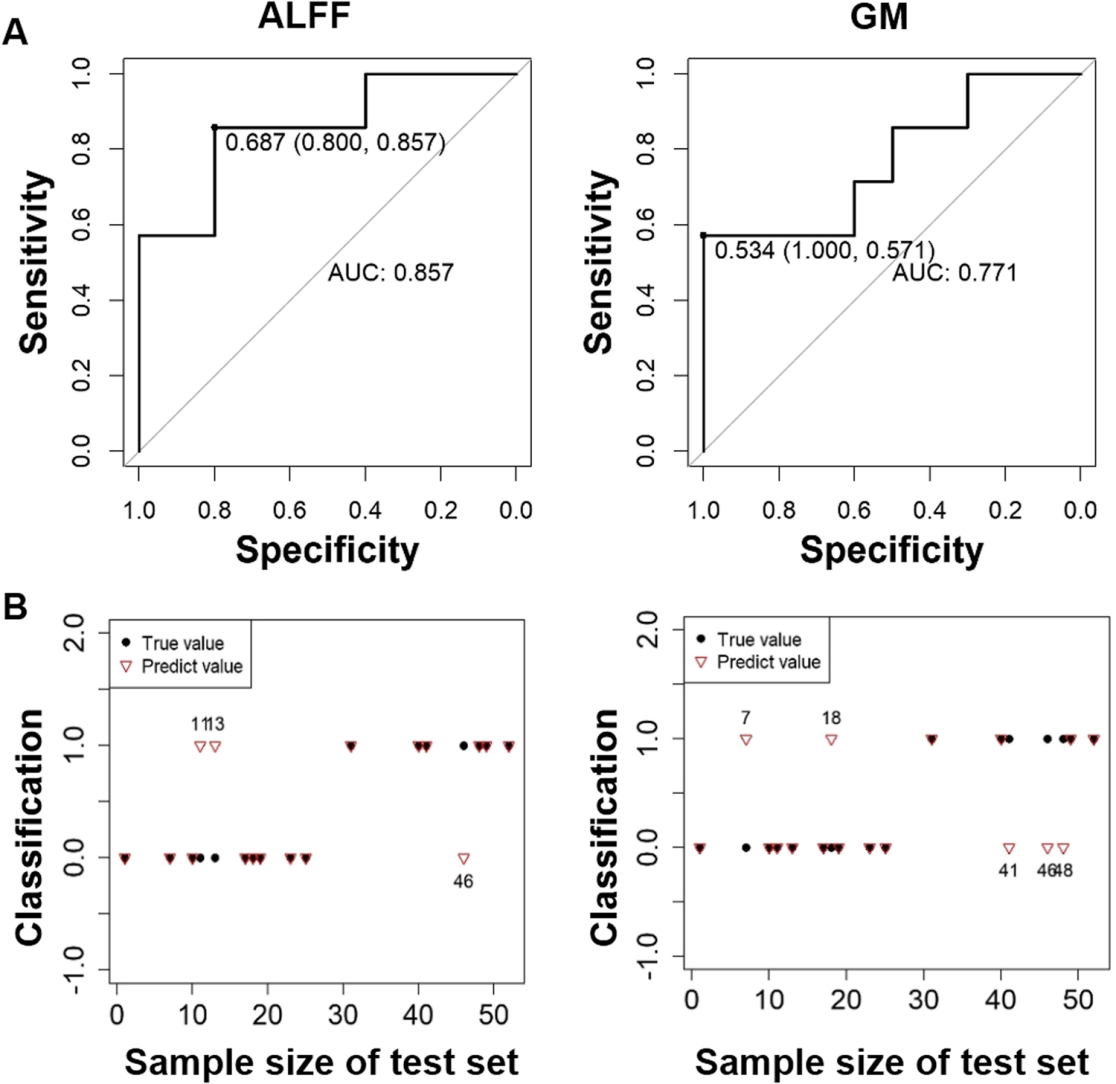
The validation of the models in predicting SZ. **A** The ROC curves show the accuracies in differentiating SZs and HCs in ALFF (85.7%) and GM (77.1%), respectively. **B** The accuracies in predicting SZ in test set for ALFF (3 participants misclassified) and GM (5 participants misclassified) analyses. Abbreviations: SZ, schizophrenia: HC, healthy control; ROC, received operation curve; GM, gray matter; ALFF, amplitude of low-frequency fluctuations; AUC, area under the curve

To examine the sensitivity and specificity of the model in predicting SZ, the training set shows the optimal prediction thresholds c* = 0.687 with accuracy 85.7% in ALFF, and the optimal prediction thresholds c* = 0.534 with 77.1% in GM (see Fig. 2A). In the test set, the prediction accuracy reached 82.4% (3 participants misclassified) in ALFF, and 76.5% (5 participants misclassified) in GM (see Fig. 2B). In Supplementary Fig. 1, additional analyses were applied to examine whether global signal regression in ALFF analysis would influence the discrimination power, and showed lower but reliable accuracy (training set: 71.4%; test set: 76.4%).

**Fig. 3:**
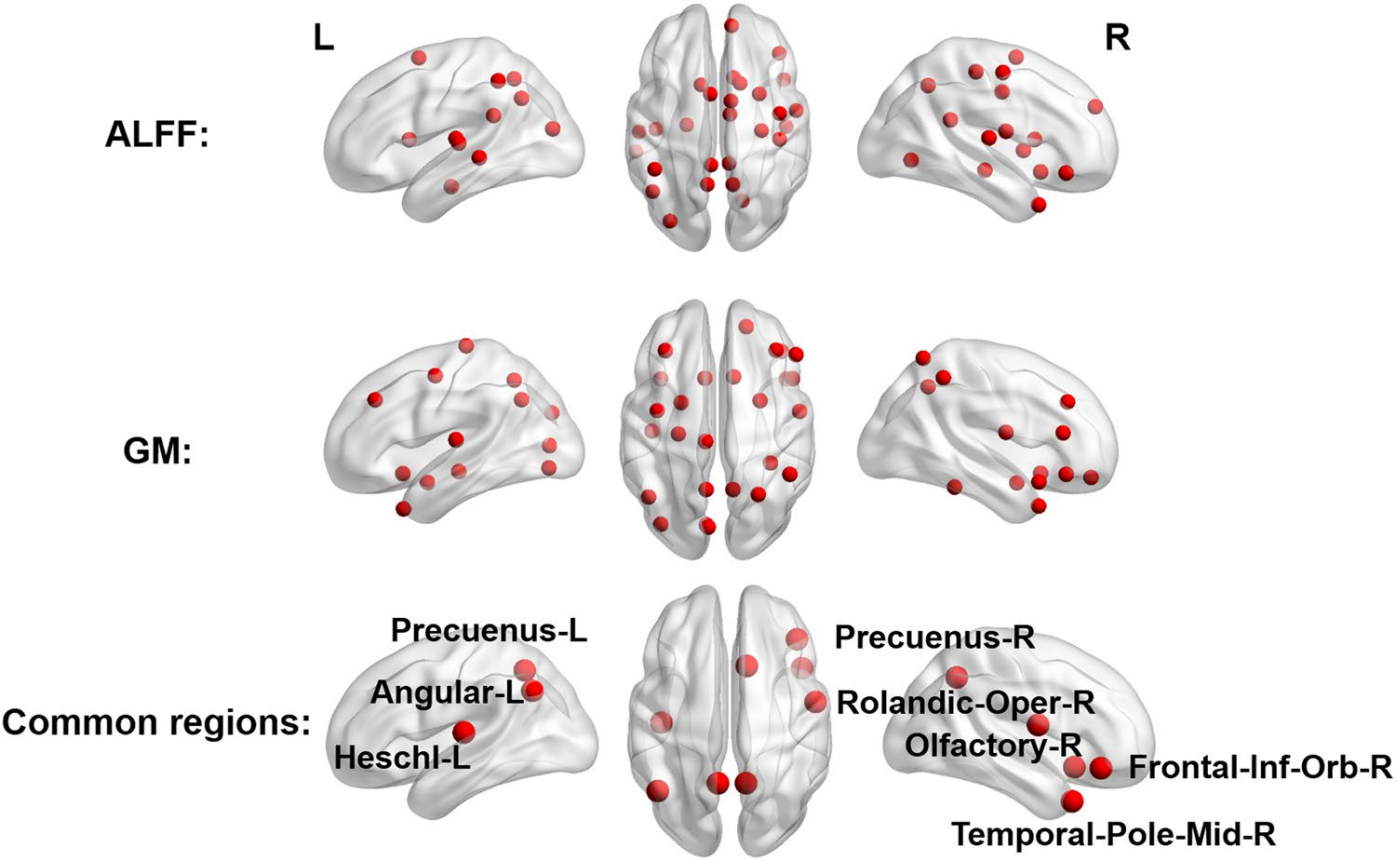
The brain regions contributing to SZ prediction in elastic net logistic regression. Twenty-seven regions were selected in the ALFF analysis, and 26 regions were selected in the GM analysis. There were 8 common regions in both two analysis. Abbreviations: SZ, schizophrenia; ALFF, amplitude of low-frequency fluctuations; GM, gray matter; L, left; R, right

Eight common brain regions were found in both ALFF and GM analyses, including the Frontal-Inf-Orb-R, Rolandic-Oper-R, Olfactory-R, Angular-L, Precuneus-L, Precuenus-R, Heschl-L, and Temporal-Pole-Mid-R. (see Table 2 and Fig. 3). Therefore, these common regions were used to examine the relationships with clinical symptoms in the following analysis.

### Correlations with PANSS

**Fig. 4:**
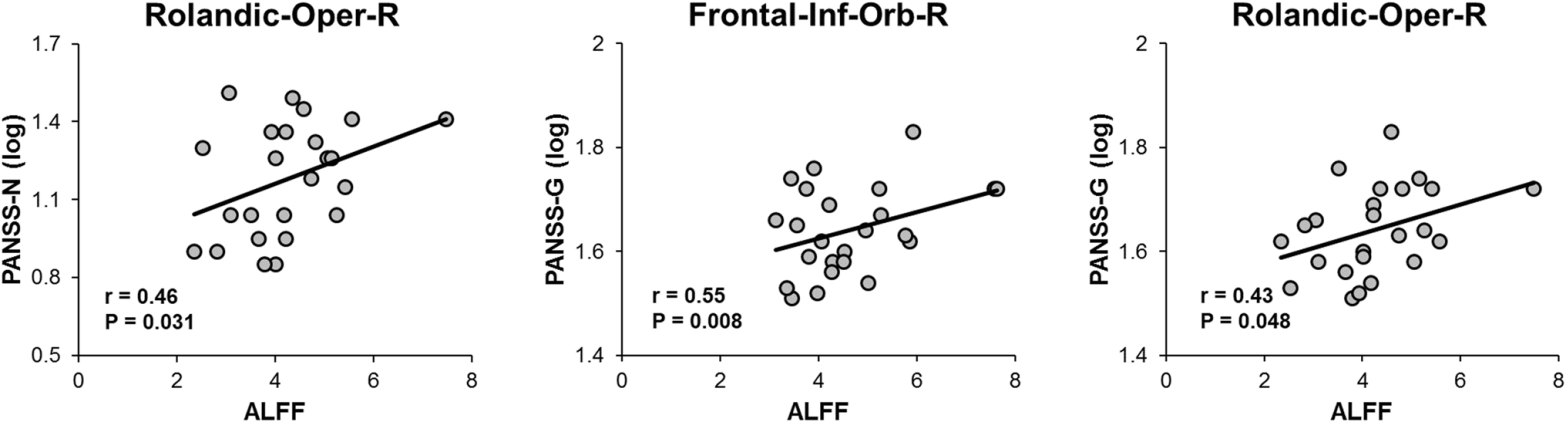
The scatterplots show the relationships between ALFF and PANSS scores in SZ patients. PANSS-N was found positively correlated with the Rolandic-Oper-R, and PANSS-G was found positively correlated with the Rolandic-Oper-R and Frontal-Inf-Orb-R. Abbreviations: SZ, schizophrenia; ALFF, amplitude of low-frequency fluctuations; PANSS-N, PANSS negative scores; PANSS-G, PANSS positive scores

The 8 common regions involved in both ALFF and GM models were selected to examine the relationship with PANSS scores. In the ALFF analysis, partial correlation analysis showed significant positive correlations between PANSS-N and the Rolandic-Oper-R (r = 0.46, *p* = 0.031), PANSS-G and the Frontal-Inf-Orb-R (r = 0.55, *p* = 0.008), PANSS-G and the Rolandic-Oper-R (r = 0.43, *p* = 0.048) (see Fig. 4). In the GM analysis, there was no significant correlation found with PANSS scores. Since lower level of education is typical in SZ patients, it may be problematic to simply control for years of education in the analysis. Thus we reanalyzed the correlation between PANSS scores and brain regions without controlling for education. Consistently, significant positive correlations were found between PANSS-G and the Frontal-Inf-Orb-R (r = 0.55, *p* = 0.006), PANSS-G and the Rolandic-Oper-R (r = 0.44, *p* = 0.047), and marginally significant correlation between PANSS-N and the Rolandic-Oper-R (r = 0.38, *p* = 0.076).

#### Discussion

In the current study, we investigated the brain functional and structural changes in female patients with SZ using elastic net logistic regression. By examining the altered ALFF and GM patterns in SZ separately, the models selected the brain regions most relevant to SZ, and showed reliable prediction accuracy in classifying patients and controls. Notably, we found 8 common regions in both ALFF and GM analyses, suggesting these regions may be core hubs in SZ-related network. In addition, the severity of SZ symptoms (PANSS) were found significantly associated with ALFF in the Frontal-Inf-Orb-R and Rolandic-Oper-R.

Since the BOLD signals are highly correlated across different brain regions, classical linear regression models are not suitable for dealing with neuroimaging data. Penalized regression has been developed to deal with multidimensional and multicollinear data by using ridge regression (Hoerl & Kennard, 1970) and the Lasso (Tibshirani, 1996), which has been demonstrated reliable and efficient in statistical analysis for neuroimaging data (Cui & Gong, 2018; Schouten et al., 2017; Scott et al., 2017). Compared with ridge and Lasso regression, elastic net regression not only reduces the dimensionality of the feature space, but also preserves meaningful correlations of the original feature space. Previous rs-fMRI studies have successfully applied elastic net penalty to do feature selection in characterizing psychiatric disorders, such as Alzheimer’s disease (Teipel et al., 2016), autism (Plitt et al., 2015), and major depression (Bhaumik et al., 2017). In resting-state functional connectivity analysis, several studies have shown that machine learning with elastic net penalty could be useful for developing diagnostic tools for SZ (Kim et al., 2016; Zhu et al., 2018). To the best our knowledge, our study applied an elastic net penalty to investigate the neural substrates of SZ by combining ALFF and GM measures for the first time. Our results demonstrated that elastic net logistic regression is a useful tool to identify the characteristics of SZ brain pattern in both functional and structural imaging data, and show reliable prediction accuracy in SZ classification (82.4% in ALFF, 76.5% in GM). Based on our results, it is worthy to validate elastic net regression by comparing with other approaches (e.g., Lasso) in future study. Notably, we also did additional analyses to examine the effect of global signal on prediction accuracy in ALFF model (Supplementary Fig. 1). After regressing out the global signal before calculating ALFF, the accuracies of identifying SZ were still reliable (training set: 71.4%; test set: 76.4%) in elastic net regression model. We speculated the lower accuracy was probably due to loss of diagnostic information in global signal. Consistently, previous studies have reported that removing global signal may lose valuable components such as potential diagnostic information in schizophrenia (Hahamy et al., 2014; Liu et al., 2017; Yang et al., 2014). Thus it should be cautious to deal with global signal and interpret the result in future fMRI research.

In the ALFF and GM analyses, 20–30 regions were selected to obtain optimal prediction accuracy, including the frontal regions, subcortical and temporal structures. Those regions are involved in several resting state brain networks, including the default mode network (DMN), executive control network and cortical-striatal network, which have been found significantly disrupted in SZ (Horga et al., 2016; Woodward et al., 2011). More importantly, 8 common regions were found in both of the two analyses, including the Frontal-Inf-Orb-R, Rolandic-Oper-R, Olfactory-R, Angular-R, Precuneus-L, Precuneus-R, Heschl-L, and Temporal-Pole-Mid-R. Guo et al. found decreased GM in the Frontal-Inf-Orb-R in first-episode SZ, which was associated with poorer cognitive functions (X. Guo et al., 2014). Another study reported significant neuroplasticity in the orbitofrontal cortex in SZ after cognitive enhancement therapy, suggesting a critical role of orbitofrontal cortex in SZ pathology (Wojtalik et al., 2015). In line with these findings, our study also showed the functional and structural changes of Frontal-Inf-Orb-R were crucial for differentiating SZs from HCs, and the ALFF value was positively associated with PANSS scores. Our results also showed the ALFF in the Rolandic-Oper-R associated with SZ pathology, suggesting the abnormality of integrating intrinsic and extrinsic signals in SZ. Consistently, previous study has reported that the Rolandic operculum was closely related to exteroceptive-interoceptive signals integration, which are necessary for self-consciousness (Blefari et al., 2017). The angular gyrus and precuneus have been found as core hubs in the DMN, which are involved in multiple cognitive functions. Taken together, converging evidence has shown that the DMN activity is enhanced in SZ patients, which is probably linked to the symptoms of ‘thought disorder’ in SZ (Hunt et al., 2017; Littow et al., 2015). In addition, our study also reported the significant contributions of the Olfactory-R, Heschl-L and Temporal-Pole-Mid-R in classifying SZ and controls, suggesting a possible explanation for perceptual distortions in SZ, such as olfactory and auditory hallucinations (Chyzhyk et al., 2015; Kiparizoska & Ikuta, 2017).

Several limitations need to be admitted in the present study. First, although the elastic net logistic regression provided promising accuracies in differentiating SZs from HCs, it should be cautious to interpret the findings using cross-validation in a small sample size (Poldrack et al., 2020). Varoquaux argued that the observed errors of cross-validation in small samples are often underestimated (Varoquaux, 2018). To confirm the model parameters were suitable, our further analyses showed tenfold cross validation giving optimal parameters compared with fivefold, 15-fold and 20-fold (Supplementary Table 1). However, a larger sample size is required to validate the findings in future research. Second, we only recruited female SZ patients in the current study. Although a number of uncertainties remain about gender differences in SZ, some previous findings have reported gender differences in neurocognitive functions and brain networks in SZ (Mendrek & Mancini-Marie, 2016). Therefore, future research need to examine whether our findings can be extended to male SZ patients. Third, previous studies have shown that SZ patients represent significant cognitive decline compared with HC. Thus more cognitive and behavioral assessments should be involved to explain the impact of SZ pathology, such as the Montreal Cognitive Assessment (MOCA) and Mini-Cog test (Tsoi et al., 2015). Lastly, some additional parameters, such as smoking and antisocial personality, may play an important role in analyzing resting-state brain patterns (Kashyap et al., 2020), which need be considered in selecting healthy control populations in the future studies.

## Conclusion

In summary, our study showed that the elastic net logistic regression could be a useful tool to identify the characteristics of SZ-induced brain deterioration for the first time. The common regions in both ALFF and GM analyses suggest that multiple brain regions play core roles in SZ-related brain networks. Our findings may help better understand the brain functional and structural changes in SZ, which provides novel insights into SZ diagnosis and prediction.

## Supplementary Information

Below is the link to the electronic supplementary material.

Supplementary file1 (DOC 586 KB)
