# Supplementary material for: Detection of functional and structural brain alterations in female schizophrenia using elastic net logistic regression
Source: Brain Imaging Behav. 2021 Jul 27;16(1):281–90. doi: 10.1007/s11682-021-00501-z (PMC8825615; doi:10.1007/s11682-021-00501-z)

**Supplementary Table 1. Cross validation in ALFF and GM analyese.**

| **Cross validation** | **Brain regions (n)** | **λ** | **Accuracy** |
| --- | --- | --- | --- |
| *ALFF model* |  |  |  |
| 5 fold | 28 | 0.031 | 0.857 |
| *10 fold | 27 | 0.038 | 0.857 |
| 15 fold | 29 | 0.030 | 0.857 |
| 20 fold | 29 | 0.025 | 0.843 |
| *GM model* |  |  |  |
| 5 fold | 8 | 0.198 | 0.60 |
| *10 fold | 26 | 0.043 | 0.771 |
| 15 fold | 16 | 0.108 | 0.614 |
| 20 fold | 0 | 0.417 | 0.50 |

Different numbers of folds in cross validation (5 fold, 10 fold, 15 fold and 20 fold) were used to examine if the selected parameters are coherent across all the different analysis. *, the optimal parameters were found using 10-fold cross validation in both ALFF and GM analyses. Abbreviations: ALFF, amplitude of low-frequency fluctuations; GM, gray matter.

**Supplementary Figure 1.** To examine the effect of global signal on prediction accuracy in ALFF model, the mean global signal was regressed out from resting-state BOLD signals before calculating ALFF. A) The parameters in logistic regression were defined using elastic net penalty. The scatterplots show the largest AUC values obtained by using 21 brain regions in ALFF model (left). The optimal λ value (λ = 1.446, α = 0.105) was determined by the minimum misclassification errors in ALFF (right). B) The ROC curve shows the optimal prediction thresholds c* = 0.578 with (71.4%) in the training set. C) The accuracy of predicting SZ was 76.5% (4 participants misclassified) in the test set. Abbreviations: ALFF, amplitude of low-frequency fluctuations; AUC, area under the curve; HC, healthy control; ROC, received operation curve; SZ, schizophrenia.


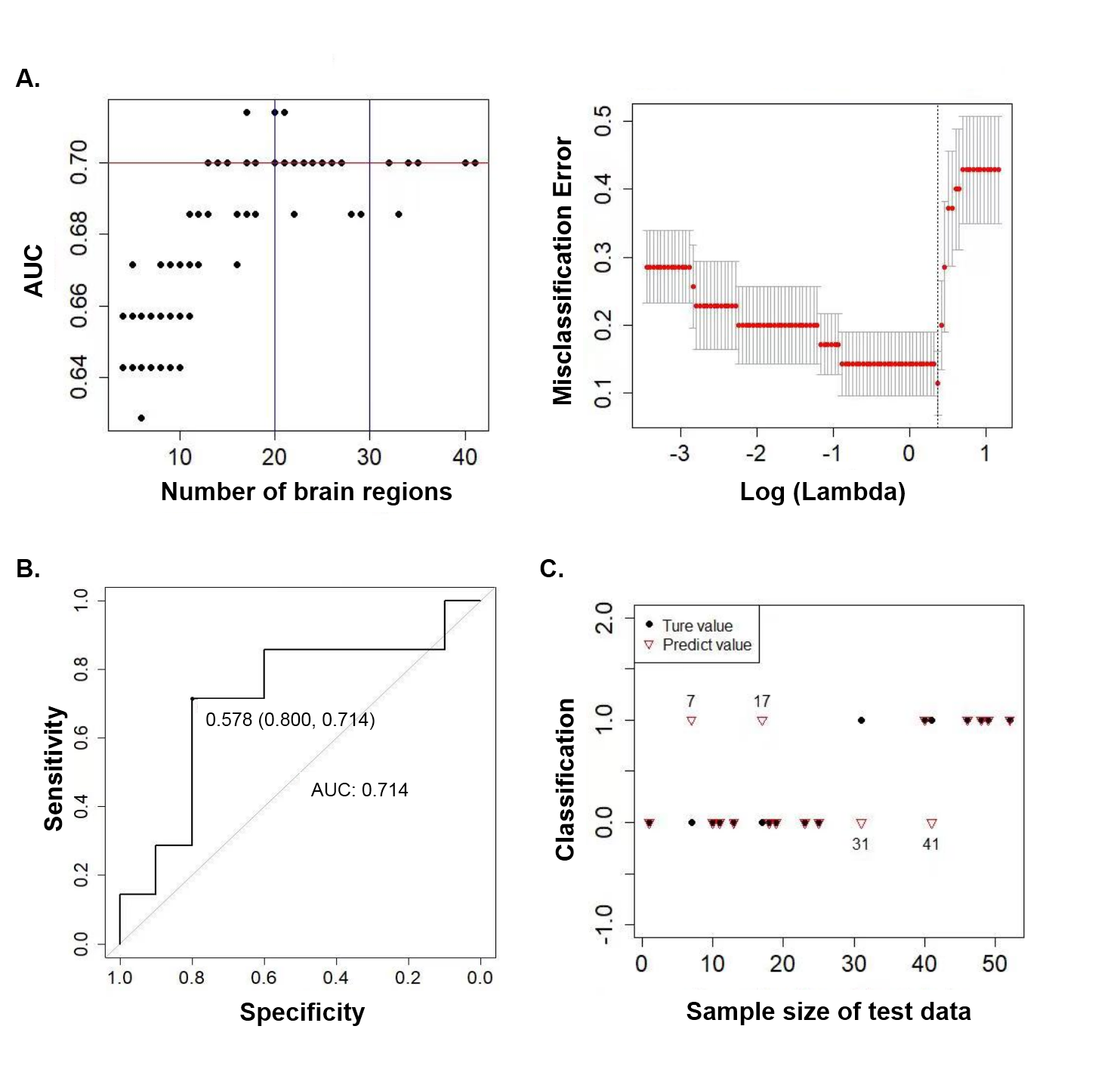

Supplement: Supplementary file 1 — Supplementary file1 (DOC 586 KB) [file 11682_2021_501_MOESM1_ESM.doc]
